# The Role of Endoscopic Ultrasound-guided Transmural Approach in the Management of Biliary Obstructions

**DOI:** 10.1097/SLE.0000000000001047

**Published:** 2022-03-25

**Authors:** Mateusz Jagielski, Michał Zieliński, Jacek Piątkowski, Marek Jackowski

**Affiliations:** Department of General, Gastroenterological and Oncological Surgery, Collegium Medicum Nicolaus Copernicus University, Toruń, Poland

**Keywords:** transmural approach, biliary obstruction, endoscopic retrograde cholangiopancreatography, endoscopic ultrasonography, transpapillary drainage, biliary drainage

## Abstract

**Background:**

Transpapillary biliary drainage in endoscopic retrograde cholangiopancreatography (ERCP) is an established method for treatment of patients with benign and malignant biliary obstruction. However, attempts to gain access to the biliary tract through the major duodenal papilla during ERCP have been unsuccessful in some patients. This study aims to determine the role of endoscopic ultrasonography (EUS)-guided transmural approach in biliary endotherapy in case of failed ERCP.

**Materials and Methods:**

A prospective analysis of the treatment outcomes of all 896 patients with obstructive jaundice secondary to biliary obstruction, who underwent endoscopic treatment in the years 2016-2021 at our institution.

**Results:**

Effective drainage of bile ducts through the major duodenal papilla during ERCP was achieved in 772/896 (86.16%) patients with biliary obstruction. In 124/896 (13.84%) patients [92 males, 32 females; mean age 63.52 (46 to 89) y] ERCP failed and EUS-guided transmural approach was performed. Benign biliary obstruction was identified in 17/124 (13.71%) patients; the remaining 107/124 (86.29%) were diagnosed with malignant biliary obstruction. EUS-guided endoscopic transpapillary biliary tract stenting with transmural access was performed in 21/124 (16.94%) patients; the remaining 103/124 (83.06%) required extra-anatomic transmural anastomosis of the bile ducts to the gastrointestinal tract. Technical success was achieved in 121/124 (97.58%) patients, while clinical success was achieved in 112/124 (90.32%). Complications were reported in 15/124 (12.1%) patients; with early complications in 12 and late complications in 3.

**Conclusions:**

Various methods of EUS-guided transmural access to bile ducts improves endotherapy outcomes of patients with biliary obstruction. Endoscopic transmural access is highly effective and associated with an acceptable number of complications.

Biliary obstruction, which is a significant disturbance in hepatic bile outflow caused by mechanical blockage, can be of either benign or malignant origin.^1^,^2^ Endoscopic retrograde cholangiopancreatography (ERCP) with biliary dilation and endoprosthesis insertion (prosthetization) is an established, minimally invasive method of treating biliary obstructions.^1^–^5^ The preferred route of access to the biliary tract is the anatomic transpapillary access gained during ERCP, which ensures the physiological drainage of bile into the duodenal lumen.^1^–^5^ However, attempts to gain access to the biliary tract through the major duodenal papilla during ERCP have been unsuccessful in some patients.^1^–^5^ In cases of ineffective transpapillary biliary drainage, percutaneous transhepatic biliary drainage and surgical drainage still serve as treatments of choice in majority of health care facilities; however, they are less effective and associated with a greater risk of complications compared with endoscopic transpapillary drainage of bile ducts.^6^,^7^

In recent years, advanced endoscopic techniques have been developed based on the use of endoscopic ultrasonography (EUS), allowing for the implementation of an extra-anatomic transmural approach (access gained through the upper gastrointestinal wall) in biliary endotherapy (ET).^8^–^10^ This provides an alternative to percutaneous or surgical drainage in cases of ERCP failure.^9^–^16^

Despite numerous studies on the transmural approach to the biliary tract, which are currently available in the literature, many aspects of this technique remain unclear. This study aims to determine the role of the extra-anatomic transmural approach in biliary ET, by analyzing its effectiveness and safety, and the application of extra-anatomic endoscopic access to bile ducts in the treatment of patients with benign and malignant biliary obstruction. This study also attempted to clarify some controversial aspects related to transmural access in biliary ET.

## MATERIALS AND METHODS

### Study Group

This is a prospective analysis of the treatment outcomes in all patients diagnosed with obstructive jaundice secondary to biliary obstruction, who underwent endoscopic treatment in the years 2016-2021 at the Department of General Surgery, Gastrointestinal Surgery and Surgical Oncology, Ludwik Rydygier Collegium Medicum in Bydgoszcz, Nicolaus Copernicus University in Toruń. After verifying all the inclusion and exclusion criteria, the final study participants consisted of patients who required EUS-guided transmural access to be obtained during ET, either due to biliary obstruction or lack of transpapillary access to bile ducts during ERCP.

All patients from the study group had preoperative imaging of abdomen (contrast-enhanced computed tomography or magnetic resonance imaging). Certainly, the results of preoperative imaging were being verified during the endoscopic procedure with use of EUS. Pathologic changes and anatomical conditions were being reassessed during EUS. As a result, therapeutic decision was taken on basis of clinical image, preoperative imaging of abdomen and confirmed during EUS procedure in every case.

### Inclusion Criteria

Patients with obstructive jaundice caused by benign or malignant biliary obstruction qualified for the study based on their clinical presentation (clinical symptoms, blood, and imaging test results), as well as the outcomes of histopathologic examination. The study included patients in whom ERCP was ineffective (3 failed ERCP attempts to catheterize bile ducts) or impossible to perform due to lack of access to the major duodenal papilla (periampullary infiltration preventing the identification of the major duodenal papilla or duodenal obstruction). The study group consisted of both female and male adult patients (18 y old and above) who provided written consent for undergoing the interventional therapy offered in this study.

### Exclusion Criteria

Patients with a history of surgical intervention in the biliopancreatic area and those who did not consent to interventional therapy were excluded from the study.

### Endoscopic Procedures

Endoscopic procedures involving the transmural approach were conducted using a therapeutic linear array echoendoscope (EG38UT Pentax, Tokyo, Japan).

Endoscopic procedures involving EUS-guided transmural access to the biliary tract commenced with the insertion of a linear echoendoscope into the upper gastrointestinal tract. Before obtaining transmural access, color flow Doppler ultrasound was performed to confirm the absence of vascular structures in the potential puncture line. Dilated intrahepatic bile ducts were punctured with a 19-G needle (EchoTip Ultra 19, Cook Medical, USA) under endosonographic guidance. After removing the stylet, bile contents were aspired to confirm the intraductal location of the needle tip. The aspirated bile was then subjected to bacteriological analysis. Next, contrast medium was injected under fluoroscopic guidance to obtain an antegrade cholangiogram. The needle was flushed with normal saline, followed by insertion of a rigid 0.035-inch guidewire (Dreamwire; Boston Scientific Corp., Marlborough, MA) into the biliary tract lumen.

### Transpapillary Bile Ducts Prosthetization Procedures Involving Transmural Approach

#### Rendezvous Technique

**FIGURE 1:**
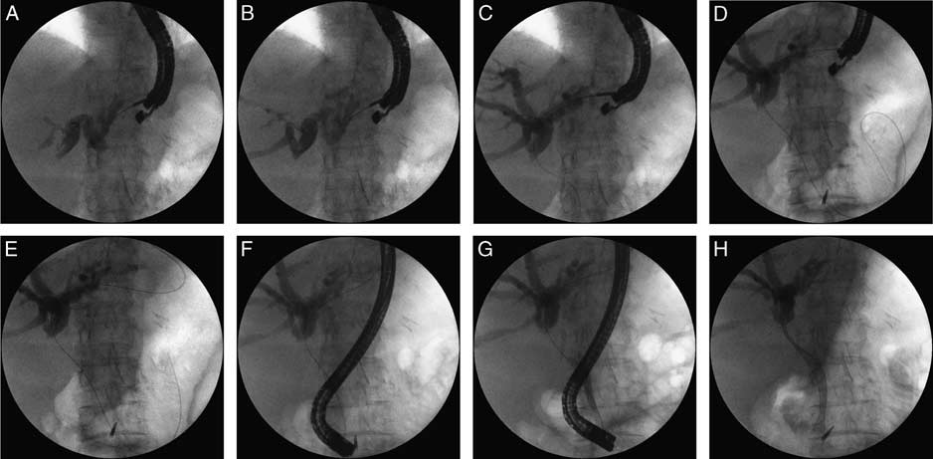
A–H, Rendezvous technique of transpapillary biliary tract stenting with the use of transmural access in the patient with an inoperable tumor of the head of pancreatic.^17^ The various steps of the procedure are shown in the figure. After transmural/transgastric puncture of intrahepatic ducts (A, B) the guidewire was introduced into the left bile duct (C) and directed toward the common bile duct, through the biliary stricture and into the duodenal lumen where the guidewire was finally looped (D, E). Duodenoscope was inserted into the duodenum and endoscopic retrograde cholangiopancreatography was then continued with insertion of partially covered self-expandable biliary prosthesis (F–H).

Transmural puncture was usually performed in the subcardia region, on the side of the lesser curvature of the stomach (or in the epicardial portion of the esophagus in cases of hypertrophy of the left lobe of the liver) where endosonographic imaging showed dilated (up to a diameter of ≥5 mm) intrahepatic bile ducts within the left lobe segments II and III (Figs. 1A–H). The guidewire was introduced into the left bile duct and directed toward the common bile duct, through the biliary stricture and into the duodenal lumen where the guidewire was finally looped. The echoendoscope was removed from the body, while maintaining the transmural/transgastric position of the guidewire in the biliary tract and duodenal lumen. Subsequently, a duodenoscope was inserted into the duodenum, and the terminal part of the guidewire located in the duodenum was grasped with rat tooth forceps or a polypectomy snare and withdrawn through the duodenoscope working channel, allowing the guidewire’s distal end to remain in the lumen of the intrahepatic bile ducts. The ERCP was then continued by performing endoscopic guidewire-assisted sphincterotomy, and tissue specimens were obtained by brushing and, whenever required, performing mechanical or pneumatic dilation of bile ducts with insertion of a fully (in benign strictures) or partially (in inoperable malignant strictures) covered self-expandable biliary prosthesis.

#### Transpapillary Bile Duct Prosthetization Via Transmural Approach Obtained With Antegrade Technique

Following transesophageal or transgastric puncture of dilated bile ducts in the left lobe of the liver and transmural insertion of the guidewire through the biliary tract into the duodenum, whenever infiltration of the peripapillary duodenal region or duodenal obstruction was diagnosed, the rendezvous method described above was not performed. Instead, transpapillary bile duct prosthetization via the transmural approach was performed using an antegrade technique. This method involves removing the needle while maintaining the transmural position of the guidewire in the biliary tract and duodenum, followed by guidewire-assisted insertion of a 10 Fr cystotome and dilation of the puncture site. Then, a fully (in benign strictures) or partially (in inoperable malignant strictures) covered self-expandable prosthesis was inserted through the dilated puncture site. Fluoroscopy-guided expansion of the endoprosthesis was performed by splinting the stricture site in such a way that one end was left in the duodenal lumen, and the other in the bile duct above the stricture level.

### Extra-anatomic Transmural Anastomosis of Bile Ducts to the Gastrointestinal Tract

#### Anastomosis of Intrahepatic Bile Ducts to the Gastrointestinal Tract^16^

**FIGURE 2:**
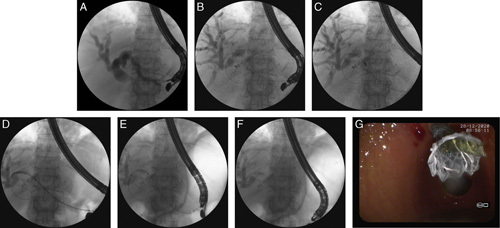
A–G, Endoscopic hepaticogastrostomy in the patient with an inoperable tumor of the major duodenal papilla and periampullary infiltration.^17^ Transmural puncture of the enlarged bile ducts within the left liver lobe was performed using a 19-G needle and a contrast agent filled the enlarged bile ducts (A). A guidewire was introduced into the left bile duct (B) and was directed toward the main bile duct. A 10 Fr cystostome was used to established a hepaticogastric fistula (C). Half-coated self-expandable endoprosthesis was introduced via the fistula (D–G).

If, despite many attempts, puncture of the dilated bile ducts (up to a diameter of ≥5 mm) in the left lobe of the liver did not allow the guidewire to be successfully passed through the stricture and placed in the duodenal lumen, an extra-anatomic anastomosis of the intrahepatic bile ducts to the gastrointestinal tract was performed. After removing the needle and securing the guidewire in the biliary lumen, guidewire-assisted cystotome insertion was performed, and a biliary-gastric (endoscopic hepaticogastrostomy) or biliary-esophageal (endoscopic hepaticoesophagostomy) fistula was constructed. Although endoscopic hepaticogastrostomy (Figs. 2A–G) was performed in most cases, patients with hypertrophy of the left lobe of the liver underwent transmural/transesophageal puncture of the dilated bile ducts in the epicardial region of the esophagus and therefore, required endoscopic hepaticoesophagostomy. A partially covered self-expandable prosthesis (Giobor, diameter of 10 mm, length of 8 or 10 cm; Taewoong Medical, Gyeonggi-do, Korea) was introduced through the transmural fistula tract formed by the cystotome in such a way that the uncovered portion of the stent was placed in the intrahepatic bile ducts. Then, a catheter was introduced through the endoprosthesis into the bile duct and a contrast agent administered for control cholangiography to verify correct placement of the transmural endoprosthesis, thereby confirming proper biliary drainage and excluding the presence of any bile leaks.

#### Anastomosis of the Extrahepatic Bile Ducts to the Gastrointestinal Tract

**FIGURE 3:**
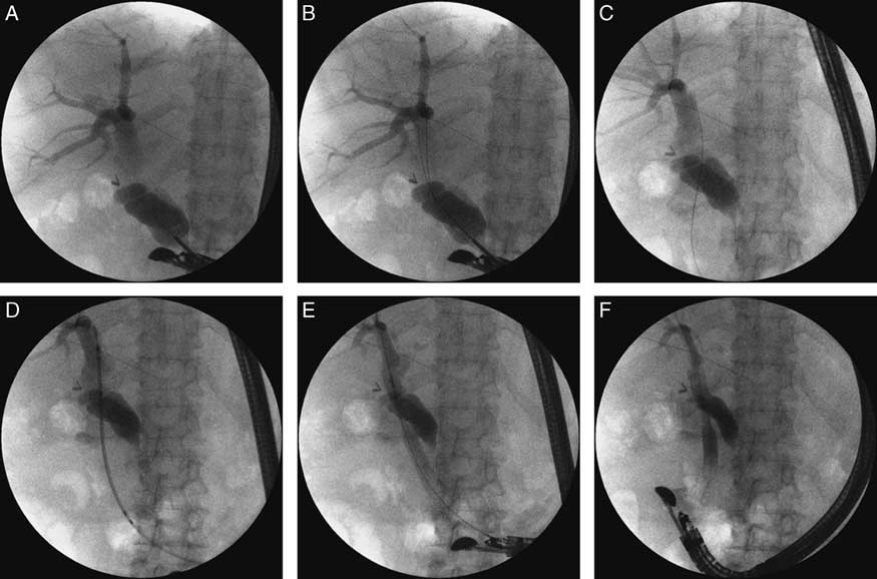
A–F, Endoscopic choledochoduodenostomy in the patient with an inoperable tumor of the head of pancreas and duodenal stenosis.^17^ Transmural/transduodenal puncture of common bile duct (A–C). A choledochoduodenal fistula was constructed using a 10 Fr cystotome. Subsequently, a partially covered biliary stent was inserted through the fistula; its distal end was placed near the hepatic hilum and the proximal end in the lumen of the duodenal bulb (D–F).

If the insertion of a linear array echoendoscope into the stomach revealed dilation of the intrahepatic bile ducts (up to a diameter <5 mm), transmural puncture of the intrahepatic ducts was not performed. Instead, an echoendoscope was introduced into the duodenal bulb to assess the width of the extrahepatic bile ducts. Whenever endosonographic imaging showed significant dilation of the common bile duct (up to a diameter ≥12 mm), transmural puncture of extrahepatic bile ducts was performed with a 19-G needle to enable further endoscopic choledochoduodenostomy. Having confirmed the position of the needle tip in the biliary lumen and administered contrast agent for cholangiography, a choledochoduodenal fistula was constructed using a 10 Fr cystotome. Subsequently, a partially covered, 60- or 80-mm-long biliary prosthesis was inserted through the fistula; its distal end was placed near the hepatic hilum and the proximal end in the lumen of the duodenal bulb. Proper drainage of the biliary tract through the newly (endoscopically) formed choledochoduodenostomy (Figs. 3A–F) was confirmed by administering contrast medium through the prosthesis into the biliary tract lumen.

If the diameter of the intrahepatic bile ducts was <5 mm and that of the common bile duct was <12 mm on endosonographic imaging and the gallbladder appeared tense and enlarged, an EUS-guided transmural puncture of the gallbladder was performed with a 19-G needle through the duodenal bulb, in order to perform endoscopic cholecystoduodenostomy. Following transduodenal puncture, the stylet was removed and the contrast administered through the needle to evaluate for cystic duct patency using fluoroscopy. If the contrast medium filled the gallbladder and then emptied freely through the cystic duct into the common bile duct, the transmural puncture could be further dilated with a 10-Fr cystotome. The resulting cholecystoduodenal fistula was then used for insertion of a self-expandable LAMS endoprosthesis. Free flow of contrast medium through the prosthesis into the duodenum and absence of leaks outside the lumen of the gastrointestinal tract confirmed proper transduodenal drainage of the gallbladder through the endoscopically created cholecystoduodenostomy.

### Definitions

Technical success was defined as successful transmural stent placement confirmed by endoscopic and radiologic imaging, such that the distal end was located within the biliary duct lumen and the proximal end within the gastrointestinal lumen.

Clinical success was defined as the clinical resolution of biliary obstruction signs, along with a decrease in cholestasis parameters observed in blood tests.

Complications of endoscopic treatment were divided into early (occurring before postoperative day 30), which were classified according to the Clavien-Dindo system,^18^ and late (occurring after postoperative day 30).

Periprocedural mortality was defined as death within 30 days of endoscopic treatment.

### Statistical Analysis

All statistical analyses were performed using R package v. 3.2.3. Quantitative variables are presented as median and interquartile range. Qualitative data are presented as numbers and percentages.

## RESULTS

A total of 896 patients diagnosed with obstructive jaundice in the course of biliary obstruction were treated endoscopically between 2016 and 2021 at the Department of General Surgery, Gastrointestinal Surgery and Surgical Oncology, Ludwik Rydygier Collegium Medicum in Bydgoszcz, Nicolaus Copernicus University in Toruń. Benign obstruction was diagnosed in 198 (22.1%) patients, and malignant obstruction in 698 (77.9%).

Effective drainage of bile ducts through the major duodenal papilla during ERCP was achieved in 772/896 (86.16%) patients with biliary obstruction.

In 124/896 (13.84%) patients [92 males, 32 females; mean age 63.52 (46 to 89) y], clinicians failed to obtain transpapillary access during ERCP. Benign biliary obstruction was identified in 17/124 (13.71%) patients; the remaining 107/124 (86.29%) were diagnosed with malignant biliary obstruction. Patient characteristics are presented in Table 1.

**TABLE 1:** The Clinical Characteristics of All 124 Patients With Biliary Obstruction Treated Endoscopically With Use of EUS-guided Transmural Approach

Male sex, n (%)	92 (74.19)
Age, mean [range]	63.52 [46-89]
Biliary obstruction cause, n (%)	
Malignant biliary obstruction cause	
Pancreatic cancer	35 (28.2)
Cholangiocarcinoma	31 (25)
Gallbladder cancer	19 (15.3)
Hepatocellular carcinoma	8 (6.48)
Major duodenal papillary cancer	6 (4.86)
Duodenal cancer	2 (1.62)
Metastatic colorectal cancer	2 (1.62)
Metastatic breast cancer	2 (1.62)
Metastatic cancer of unknown origin	2 (1.62)
Benign biliary obstruction cause	
Chronic pancreatitis	13 (10.44)
Postoperative injury after cholecystectomy	2 (1.62)
Mirizzi syndrome	1 (0.81)
Idiopathic	1 (0.81)
Suppurative cholangitis, n (%)	44 (35.48)
Reason for endoscopic ultrasonography-guided transmural biliary approach, n (%)	
Duodenal obstruction	43 (34.67)
Periampullary infiltration	66 (53.23)
Failed biliary cannulation	11 (8.87)
Surgically altered anatomy	4 (3.23)

EUS-guided endoscopic transpapillary biliary tract stenting with transmural access was performed in 21/124 (16.94%) patients; the remaining 103/124 (83.06%) required extra-anatomic transmural anastomosis of the bile ducts to the gastrointestinal tract.

Technical success was achieved in 121/124 (97.58%) patients, while clinical success was achieved in 112/124 (90.32%). Complications were reported in 15/124 (12.1%) patients; with early complications in 12 (Clavien-Dindo grade II, 6 patients; Clavien-Dindo grade III, 2 patients; Clavien-Dindo grade V, 4 patients), and late complications in 3.

Average follow-up was 9 (3 to 28) months. None of 124 patients from the study underwent surgery due to primary disease causing biliary tract stricture. In case of benign strictures endoscopic transmural anastomosis of the bile ducts to the gastrointestinal tract was sufficient. In case of malignant strictures, the changes were inoperable in the moment of endoscopic procedure of transmural biliary drainage. Despite used chemotherapy none of the patients with malignant obstruction was qualified for surgical resection after oncological treatment.

### Transpapillary Biliary Tract Stenting With Transmural Access

Endoscopic transpapillary stenting procedures with transmural access were performed in 21/124 (16.94%) patients [19 men, 2 women; mean age 58.16 (46 to 71) y]. While the rendezvous technique (using the transmural approach) with transpapillary biliary stenting (Figs. 1A–H) was performed in 20/124 (16.13%) patients, only 1/124 (0.81%) patient underwent transpapillary biliary stenting with transmural access (antegrade technique).

Benign biliary obstruction was the cause of obstructive jaundice in 15 patients, while the other 6 had malignant biliary obstruction.

ERCP was ineffective due to peripapillary infiltration, preventing the identification of the major duodenal papilla in 16 patients. The insertion of the duodenoscope was deemed impossible in three patients due to duodenal obstruction. Surgically altered anatomy of the upper gastrointestinal tract was observed in 2 patients.

Technical success was confirmed in 21/21 (100%) patients, while clinical success was observed in 19/21 (90.48%).

Complications of endoscopic treatment were recorded in 2/21 (9.52%) patients. Both patients experienced an early complication in the form of upper gastrointestinal bleeding. One patient required transfusion of blood and blood products (Clavien-Dindo grade II), while the other not only required transfusion of blood and blood products but also qualified for endoscopic management of bleeding (Clavien-Dindo grade III) with hemostatic clips; access was obtained from the transmural gastric puncture site. No fatal complications were observed.

No late complications were observed in this group of patients.

### Extra-anatomic Transmural Anastomosis of the Biliary and Gastrointestinal Tracts

#### Anastomosis of Intrahepatic Biliary Ducts to the Gastrointestinal Tract

Extra-anatomic transmural anastomosis of the intrahepatic bile ducts to the gastrointestinal tract was performed in 87/124 (70.16%) patients [61 men, 26 women; mean age 72.59 (55 to 89) y], 84/124 (67.74%) of which underwent anastomosis of the intrahepatic bile ducts within the left lobe of the liver to the stomach (endoscopic hepaticogastrostomy) (Figs. 2A–G). The remaining 3/124 (2.42%) patients required anastomosis of intrahepatic bile ducts within the left lobe of the liver to the esophagus (endoscopic hepaticoesophagostomy).

Obstructive jaundice was caused by a benign biliary obstruction in one patient, and by malignant biliary obstruction in 86 patients.

In this group of patients, ERCP failed due to unsuccessful placement of biliary tract catheter despite repeated (3) attempts (7 patients) or the inability to perform ERCP (duodenal obstruction in 36 patients, infiltration of the duodenal wall in the peripapillary region preventing identification of the major duodenal papilla in 42 patients, and surgically altered anatomy of the upper gastrointestinal tract in 2 patients).

Technical success was achieved in 85/87 (97.7%) patients, whereas clinical success was recorded in 78/87 (89.66%).

Complications of endoscopic treatment were observed in 12/87 (13.79%) patients. Early postoperative complications were reported in nine patients. Bleeding into the upper gastrointestinal tract requiring conservative treatment with transfusion of packed red blood cells and fresh frozen plasma (Clavien-Dindo grade II), was observed in 3 patients. Another 2 patients presented signs of biliary sepsis requiring intravenous broad-spectrum antibiotic therapy (Clavien-Dindo grade II). The periprocedural mortality (Clavien-Dindo grade V) occurred in 4/87 (4.6%) patients, 3 of whom died due to biliary peritonitis caused by bile leakage from an ineffective endoscopic biliary-gastric anastomosis. One patient died of biliary sepsis.

Late postoperative complications manifesting as obstruction of the transmural stent were observed in 3 patients. Late transmural stent migration complicated by a biliary-pleural fistula was diagnosed in one patient who underwent endoscopic hepaticoesophagostomy.^19^

#### Anastomosis of Extrahepatic Bile Ducts to the Gastrointestinal Tract

Endoscopic extra-anatomic anastomosis of the extrahepatic bile ducts to the gastrointestinal tract was performed in 16/124 (12.91%) patients [12 men, 4 women; mean age 70.89 (58 to 81 y)]. In 15/124 (12.1%) of these patients, anastomosis of the common bile duct to the duodenum (endoscopic choledochoduodenostomy) (Figs. 3A–F) was performed, whereas 1/124 (0.81%) patient required anastomosis of the gallbladder to the duodenum (endoscopic cholecystoduodenostomy).

One patient was diagnosed with benign biliary obstruction and 15 with malignant biliary obstruction.

The reasons for ERCP failure in this group of patients include unsuccessful placement of the biliary tract catheter after 3 attempts at ERCP (4 patients), and the inability to perform ERCP (duodenal obstruction in 4 patients, infiltration of the duodenal wall in the peripapillary region preventing identification of the major duodenal papilla in 8 patients).

Technical success of endoscopic treatment was achieved in 15/16 (93.75%) patients, and clinical success in 15/16 (93.75%).

Early complications of endoscopic treatment were diagnosed in 1/16 (6.25%) patient who required surgical intervention (Clavien-Dindo grade III) due to complete dehiscence of the cholecysto-duodenal anastomosis caused by transmural stent migration. No fatal complications were observed.

No late complications of endoscopic treatment were observed in this group of patients.

## DISCUSSION

The most preferred and physiological method of draining bile into the gastrointestinal tract is transpapillary drainage,^1^–^5^ which despite several failed ERCP attempts, was achieved in 21 patients by means of transgastric puncturing of intrahepatic bile ducts with the use of EUS-guided transmural access. Transmural access combined with rendezvous or antegrade techniques improves the effectiveness of anatomical transpapillary drainage in patients with biliary obstruction by ensuring highly efficient drainage (thought to be due to the unchanged physiological route of bile outflow into the duodenum).^11^–^15^,^20^–^22^ However, it needs to be emphasized that endoscopic procedures that involve transmural access for transpapillary biliary stenting (the rendezvous and antegrade techniques) are technically challenging even for clinicians highly specialized in interventional EUS treatment. The most frequently occurring difficulty in this type of procedure is the inability to pass the guidewire through the biliary stricture and introduce it to the duodenum. In this study, each extra-anatomic transmural puncture of the bile ducts involved attempts at transpapillary guidewire insertion through the bile ducts into the duodenum. Extra-anatomic anastomosis of the bile ducts to the gastrointestinal lumen were performed only when attempts to introduce the guidewire by transmural puncture into the duodenum were ineffective.

No clear guidelines regarding the choice of transmural puncture site in cases of extra-anatomic access to bile ducts are currently available in the literature. The choice of puncture site should certainly depend on anatomic conditions, location of the stricture, primary medical condition responsible for the obstruction, and evaluation of the possible risk of complications. The clinical experience of a health care provider is an important factor.^23^ Although many authors consider extrahepatic access to be technically easier, recent studies do not show significant differences in terms of treatment efficacy and safety between extrahepatic and intrahepatic transmural endoscopic drainage.^24^ Previous studies have shown that the most preferred method of transmural access for bile duct drainage is transduodenal access by endoscopic choledochoduodenostomy, as it is characterized by conditions that are most similar to transpapillary access, which ensures physiological outflow of bile into the duodenum.^11^–^14^ As presented above, approaches most frequently implemented at our health care facility involve transgastric access to the intrahepatic portion of the biliary tract. First, this approach provides the best chances for introduction of a guidewire through bile ducts into the duodenum and ensuring physiological transpapillary access. Second, should the attempts to achieve transpapillary drainage via transmural access fail, endoscopic anastomosis of intrahepatic bile ducts to the gastrointestinal tract (endoscopic hepaticoesophagostomy and hepaticogastrostomy) are characterized by the widest range of indications, especially in cases where performing another anastomosis of the bile duct to the gastrointestinal tract is impossible (usually in those with tumors infiltrating the hepatic hilum or other obstructions of intrahepatic bile ducts).^25^–^27^ Moreover, neither duodenal obstruction nor surgically altered anatomy of the upper gastrointestinal tract is a contraindication for transmural access to the intrahepatic portion of the biliary tract.^25^–^27^ Ogura et al,^21^ observed that obtaining access to the intrahepatic biliary tract by means of gastric puncture seems reasonable in distal malignant strictures of the common bile duct, as extra-anatomic transmural drainage of the intrahepatic biliary tract prevents overgrowth of the transmural stent from contact between the endoprosthesis and tumor tissue, which in turn allows for a longer period of endoprosthesis patency. Furthermore, publications available in the literature demonstrate similar rates of treatment effectiveness in endoscopic transmural drainage of intrahepatic and extrahepatic biliary tracts,^28^ while also emphasizing a higher risk of serious complications such as bile leak into the peritoneal cavity associated with the use of extrahepatic access; this also supports transmural access to the extrahepatic bile ducts.^29^

In this study, extra-anatomic transmural drainage of extrahepatic bile ducts (endoscopic choledochoduodenostomy) was performed only when anastomosis of the intrahepatic biliary tract to the gastrointestinal lumen was impossible because of the small diameter of the intrahepatic bile ducts. On the other hand, extrahepatic access in the form of endoscopic cholecystoduodenostomy was used as a last-resort in cases where no other transmural endoscopic access to bile ducts was possible. Endoscopic cholecystoduodenostomy is associated with a high risk of complications due to anatomical conditions. The cholestatic gallbladder observed in patients with obstructive jaundice often appears tense and thin-walled. Puncture of the cholestatic gallbladder and drainage by decompression distances the gastrointestinal wall from the gallbladder, resulting in partial leakage or complete dehiscence of endoscopic cholecystoduodenal anastomosis and acute biliary peritonitis. Another problem associated with endoscopic cholecystoduodenostomy is cystic duct obstruction. Endoscopic cholecystoduodenostomy may be applied in biliary tract drainage only if the cystic duct is patent; otherwise, biliary drainage through the cholecystoduodenal anastomosis is ineffective.

Although endoscopic transmural (internal) drainage presents similar rates of technical and clinical success as percutaneous (external) drainage, it is associated with a significantly lower risk of complications, especially those of infectious origin.^6^,^7^ In most centers, percutaneous drainage remains the only alternative in cases of ERCP failure.^6^,^7^ Since extra-anatomic transmural techniques for gaining access to bile ducts in biliary endoscopic therapy were introduced at our center, no patient has needed percutaneous transhepatic biliary drainage. The implementation of endoscopic transmural drainage of bile ducts at our center has limited the use of other biliary drainage techniques in cases of ERCP failure.

The results presented in the study suggest that some modifications of the intervention algorithm in case of transpapillary drainage failure during ERCP in patients diagnosed with obstructive jaundice should be considered.

The main limitations of the study include the lack of randomization and the fact that the study was carried out on a selected group of patients treated at only one center.

In conclusion, this study demonstrated the constant development of various methods of EUS-guided endoscopic transmural access to bile ducts, starting with techniques that provide anatomic transpapillary drainage (rendezvous or antegrade techniques) and ending with an extra-anatomic anastomosis of bile ducts to the gastrointestinal tract which improves treatment outcomes of patients with biliary obstruction. If traditional transpapillary biliary drainage is ineffective, endoscopic techniques based on transmural access will improve ET outcomes while reducing the need for other biliary drainage techniques.
